# Transspinal Direct Current Stimulation Produces Persistent Plasticity in Human Motor Pathways

**DOI:** 10.1038/s41598-017-18872-z

**Published:** 2018-01-15

**Authors:** Lynda M. Murray, Behdad Tahayori, Maria Knikou

**Affiliations:** 1 Motor Control and NeuroRecovery Research Laboratory (Klab4Recovery), Department of Physical Therapy, City University of New York, College of Staten Island, New York, NY 10314 USA; 2 PhD Program in Biology and Neuroscience, The Graduate Center, City University of New York, New York, NY 10016 USA

## Abstract

The spinal cord is an integration center for descending, ascending, and segmental neural signals. Noninvasive transspinal stimulation may thus constitute an effective method for concomitant modulation of local and distal neural circuits. In this study, we established changes in cortical excitability and input/output function of corticospinal and spinal neural circuits before, at 0–15 and at 30–45 minutes after cathodal, anodal, and sham transspinal direct current stimulation (tsDCS) to the thoracic region in healthy individuals. We found that intracortical inhibition was different among stimulation polarities, however remained unchanged over time. Intracortical facilitation increased after cathodal and anodal tsDCS delivered with subjects seated, and decreased after cathodal tsDCS delivered with subjects lying supine. Both cathodal and anodal tsDCS increased corticospinal excitability, yet facilitation was larger and persisted for 30 minutes post stimulation only when cathodal tsDCS was delivered with subjects lying supine. Spinal input/output reflex function was decreased by cathodal and not anodal tsDCS. These changes may be attributed to altered spontaneous neural activity and membrane potentials of corticomotoneuronal cells by tsDCS involving similar mechanisms to those mediating motor learning. Our findings indicate that thoracic tsDCS has the ability to concomitantly alter cortical, corticospinal, and spinal motor output in humans.

## Introduction

One method to induce neuromodulation via stimulation in humans is by weak direct current delivered through the head or vertebral column^1^. Transcortical direct current stimulation (tDCS) alters corticospinal excitability in a polarity-specific manner, with cathodal decreasing and anodal increasing transcranial magnetic stimulation (TMS)-induced motor evoked potentials (MEPs) recorded from arm and leg muscles^2,3^. In addition, tDCS alters cerebello-brain inhibition^4^, shifts intracortical facilitation towards inhibition^5^, increases excitability of the supplementary motor area^6^, and potentiates reciprocal postsynaptic inhibition in arm and leg antagonistic muscles^7,8^. It has been suggested that tDCS-mediated plasticity involves long-term potentiation and long-term depression like mechanisms via modification of N-methyl-D-aspartate receptors^9^.

Direct current delivered transcutaneously to the spine (termed here transspinal direct current stimulation; tsDCS) is a relatively new neuromodulatory method. tsDCS modulates the cortical silent period and corticospinal excitability of distal toe muscles, produces short- and long-term changes in the excitability of the cortico-phrenic and lemniscal pathways, and alters spinal processing of nociceptive inputs in healthy humans^10–13^. Reports on changes in soleus H-reflex excitability are somewhat controversial, with no changes or marginal changes in the ratio of maximal H-reflex and M-wave (Hmax/Mmax)^13–15^, while motor unit recruitment improves in a polarity-specific manner in healthy humans^16^. Changes in synaptic transmission by tsDCS have also been assessed in both human and animal. Specifically, cathodal tsDCS increases and anodal tsDCS decreases the human soleus H-reflex low-frequency stimulation induced homosynaptic depression^14^, consistent with findings reported after local stimulation but not after tsDCS in anaesthetized rats^17^. Last, tsDCS affects spinal motor output by enabling the depolarized group Ia afferents to evoke larger monosynaptic excitatory postsynaptic potentials onto alpha motoneurons^18^.

Because the spinal cord is an integration center for descending, ascending, and segmental neural signals, transspinal stimulation may constitute an effective method for concomitant modulation of local and distal neural circuits. We hypothesized that tsDCS modulates cortical excitability, and corticospinal - spinal input/output function in a polarity-specific manner. This hypothesis is appealing because demonstration of neuromodulation at sites distal from the stimulating electrode would be applicable to many different types of neurological disorders that the function of cortical and corticospinal neural circuits is significantly impaired. We further hypothesized that the neuromodulatory effects of tsDCS depend on body position. The rationale for this hypothesis was based on the fact that the neurophysiological characteristics (threshold, latency, and duration) and susceptibility of the transspinal evoked potentials (TEPs) recorded simultaneously from leg muscles to spinal inhibition depends on body position^19–22^. The supine position appears to be optimal for TEPs elicitation, possibly due to changes in spinal curvature and surrounding soft tissue, while different neural structures are stimulated with subjects seated, prone, semi-prone, and supine^22^. To test our hypotheses, we assessed the immediate and after-effects of tsDCS on cortical feedback mechanisms, and corticospinal-spinal input/output function in healthy individuals. Subjects were blind to the polarity of stimulation and randomly assigned to receive cathodal, anodal, or sham tsDCS for 30 minutes while seated or cathodal tsDCS while lying supine.

## Results

**Figure 1:**
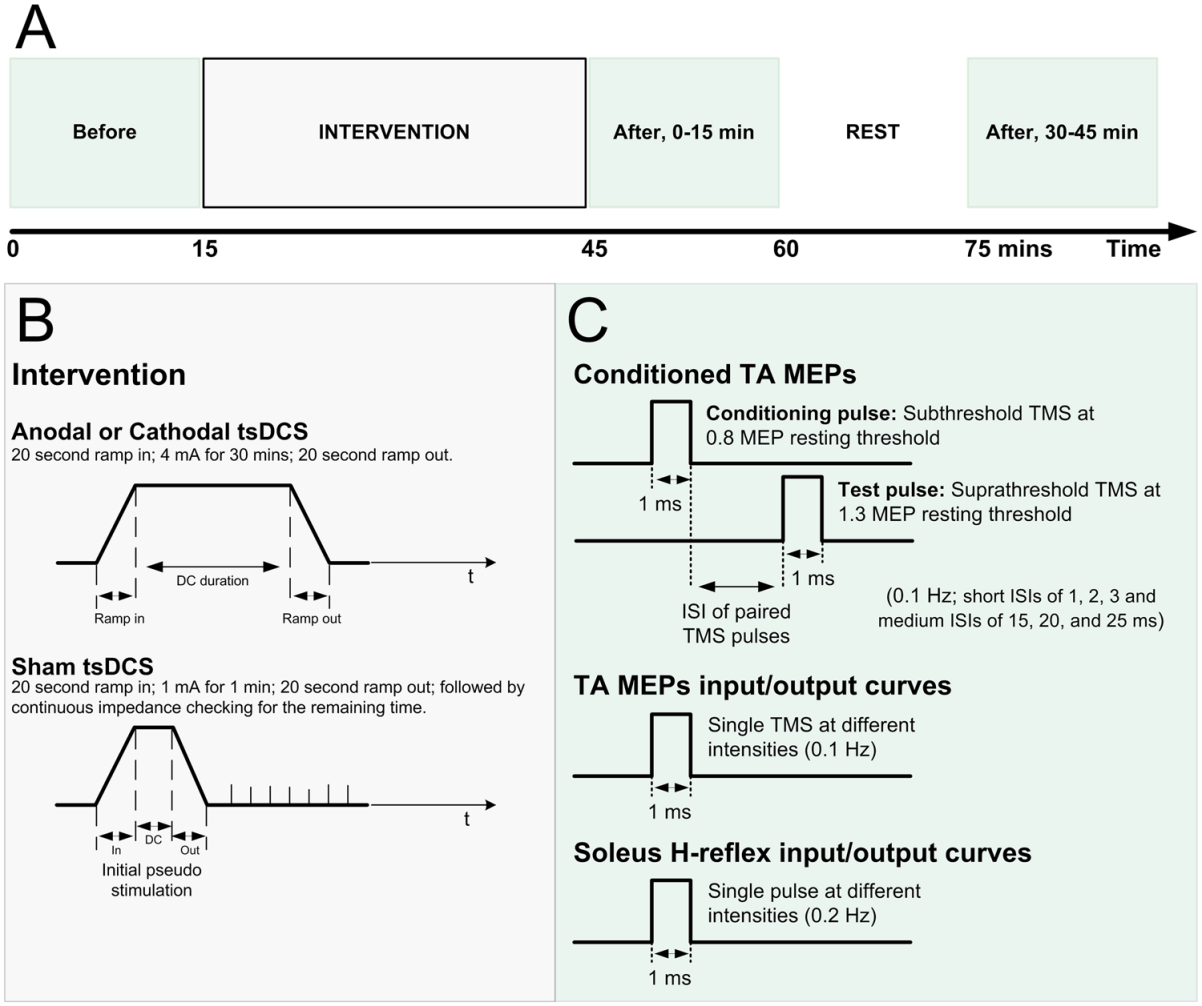
Stimulation and experimental schematic. (**A**) Transspinal direct current stimulation (tsDCS) protocol. (**B**) Illustration of anodal, cathodal and sham tsDCS delivered during the intervention. (**C**) Illustration of paired and single transcranial magnetic stimulation (TMS) pulses for recording tibialis anterior motor evoked potentials (TA MEPs) as well as transcutaneous electrical stimulation of the posterior tibial nerve for soleus H-reflexes and M-waves. Paired TMS pulses were used to condition TA MEPs at short interstimulus intervals (ISIs) of 1, 2, 3 and medium ISIs of 15, 20 and 25 ms. Single-pulse TMS and transcutaneous electrical stimulation at different stimulation intensities was delivered for assembling the TA MEP and soleus H-reflex input/output curves, respectively.

Twenty-two healthy subjects (27.9 ± 9.6, mean age yrs ± SD; 10 female) participated, and a total of 42 experiments were completed. The experimental protocols for neurophysiological outcome measures and stimulation characteristics are depicted in Fig. 1. tsDCS over the thoracic spine was well tolerated by all subjects and no adverse events were reported during or after termination of the study. All subjects reported tingling or itching sensations during but not after cessation of stimulation.

The tibialis anterior (TA) MEP latency was estimated from the unconditioned TA MEPs recorded at 1.2–1.3 MEP resting threshold. The TA MEPs latency remained unchanged following cathodal (before: 30.8 ± 1.76 ms, 0–15 min after: 30.62 ± 1.59 ms, 30–45 min after: 30.68 ± 1.56 ms; mean ± SD) and anodal (before: 30.31 ± 2.1 ms, 0–15 min after: 30.21 ± 2.14 ms, 30–45 min after: 30.16 ± 1.9 ms) tsDCS [two-way repeated measures analysis of variance (rmANOVA) with factors time: F_(2)_ = 0.05, *p* = 0.94 and polarity: F_(1)_ = 1.44, *p* = 0.23].

### Changes in Cortical Excitability by tsDCS

**Figure 2:**
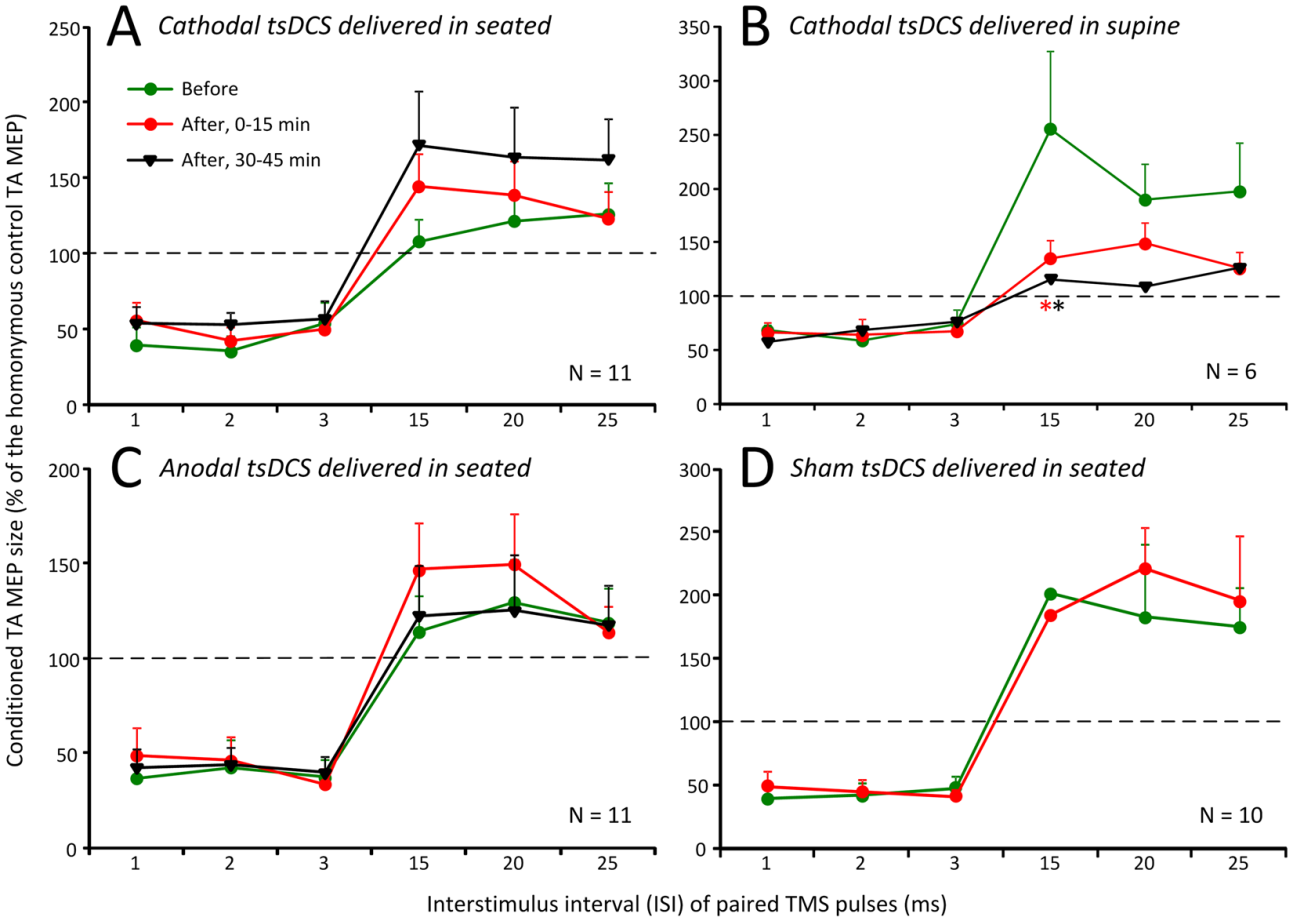
Cortical feedback mechanisms before and after transpinal direct current stimulation (tsDCS). Overall amplitude of tibialis anterior motor evoked potentials (TA MEPs) conditioned by subthreshold transcranial magnetic stimulation (TMS) before (green circles), 0–15 min after (red circles), and 30–45 min after (black triangles) 30 minutes of tsDCS. Cathodal (**A**), anodal (**C**), and sham (**D**) stimulation was delivered whilst the subjects were seated and cathodal tsDCS (**B**) was delivered with the subjects lying supine. Error bars indicate SE.

Changes in excitability of cortical circuits were assessed via paired TMS pulses delivered to the left primary motor cortex at short (1, 2, and 3 ms) and medium (15, 20, and 25 ms) interstimulus intervals (ISIs). Short-latency ISIs were used to test intracortical inhibition whilst medium-latency ISIs were used to test intracortical facilitation. The amplitude of the conditioned TA MEPs, normalized to the unconditioned TA MEP for each stimulation polarity and all ISIs tested, are indicated in Fig. 2. Two-way rmANOVA with factors time (before, 0–15 min, 30–45 min) and stimulation protocol (cathodal seated, cathodal supine, anodal seated, and sham seated) with conditioned TA MEPs grouped based on ISI showed a significant effect among stimulation protocols on the conditioned TA MEPs at short ISIs (F_(3)_ = 6.6, *p* < 0.001) but not a significant effect across time (F_(2)_ = 0.53, *p* = 0.58). Holm-Sidak pairwise multiple comparisons for stimulation protocols showed that the conditioned TA MEPs in the supine cathodal versus seated cathodal tsDCS (t = 3.12, *p* = 0.002), supine cathodal versus sham tsDCS (t = 3.75, *p* < 0.001), and cathodal supine versus anodal seated tsDCS (t = 4.31, *p* < 0.001) were significantly different. Non-significant differences were found for cathodal seated versus anodal seated, cathodal seated versus sham, and sham versus anodal seated (all *p* > 0.05). These findings suggest that the strength of intracortical inhibition was different among stimulation protocols, however remained unchanged over time.

With respect to intracortical facilitation, two-way rmANOVA with factors time (before, 0–15 min, 30–45 min) and stimulation protocol (cathodal seated, cathodal supine, anodal seated, and sham seated) with conditioned TA MEPs grouped based on ISI showed a non-significant effect across time (F_(2)_ = 0.49, *p* = 0.62), and a significant effect among stimulation protocols (F_(3)_ = 9.1, *p* < 0.001) on the conditioned TA MEPs at medium ISIs. Holm-Sidak pairwise multiple comparisons for stimulation protocols showed that the conditioned TA MEPs during the sham protocol were significantly different from all other experimental protocols tested (*p* < 0.05). Further, a significant interaction between time and stimulation protocol was found (F_(6)_ = 2.68, *p* = 0.015), suggesting that the effects across time depended on the stimulation protocol. Specifically, Holm-Sidak pairwise multiple comparisons showed no significant differences of the conditioned TA MEPs across time for cathodal seated and anodal seated (*p* > 0.05), while a significant effect was found in the supine cathodal tsDCS across time (before versus 0–15 min: *p* = 0.007; before versus 30–45 min: *p* = 0.003). Furthermore, two-way rmANOVA during which sham stimulation protocol was not included showed that at 0–15 min the conditioned TA MEPs in the cathodal seated tsDCS were different from the cathodal supine tsDCS (*p* < 0.001), and the conditioned TA MEPs in the cathodal seated tsDCS were different from the anodal seated tsDCS (*p* < 0.001). Holm-Sidak pairwise multiple comparisons showed that for within cathodal seated tsDCS, the conditioned MEPs were increased at 0–15 min compared to before (*p* < 0.001), and further increased at 30–45 min as compared to 0–15 min (*p* < 0.001). Further, changes over time for the anodal seated tsDCS were found between 0–15 min and before (*p* < 0.034), while at 30 min intracortical facilitation returned to baseline amplitudes. Together, these results suggest that intracortical facilitation was increased after cathodal and anodal tsDCS delivered with subjects seated, and was decreased after cathodal tsDCS delivered with subjects lying supine.

### Changes in Corticospinal Input/Output Function by tsDCS

**Figure 3:**
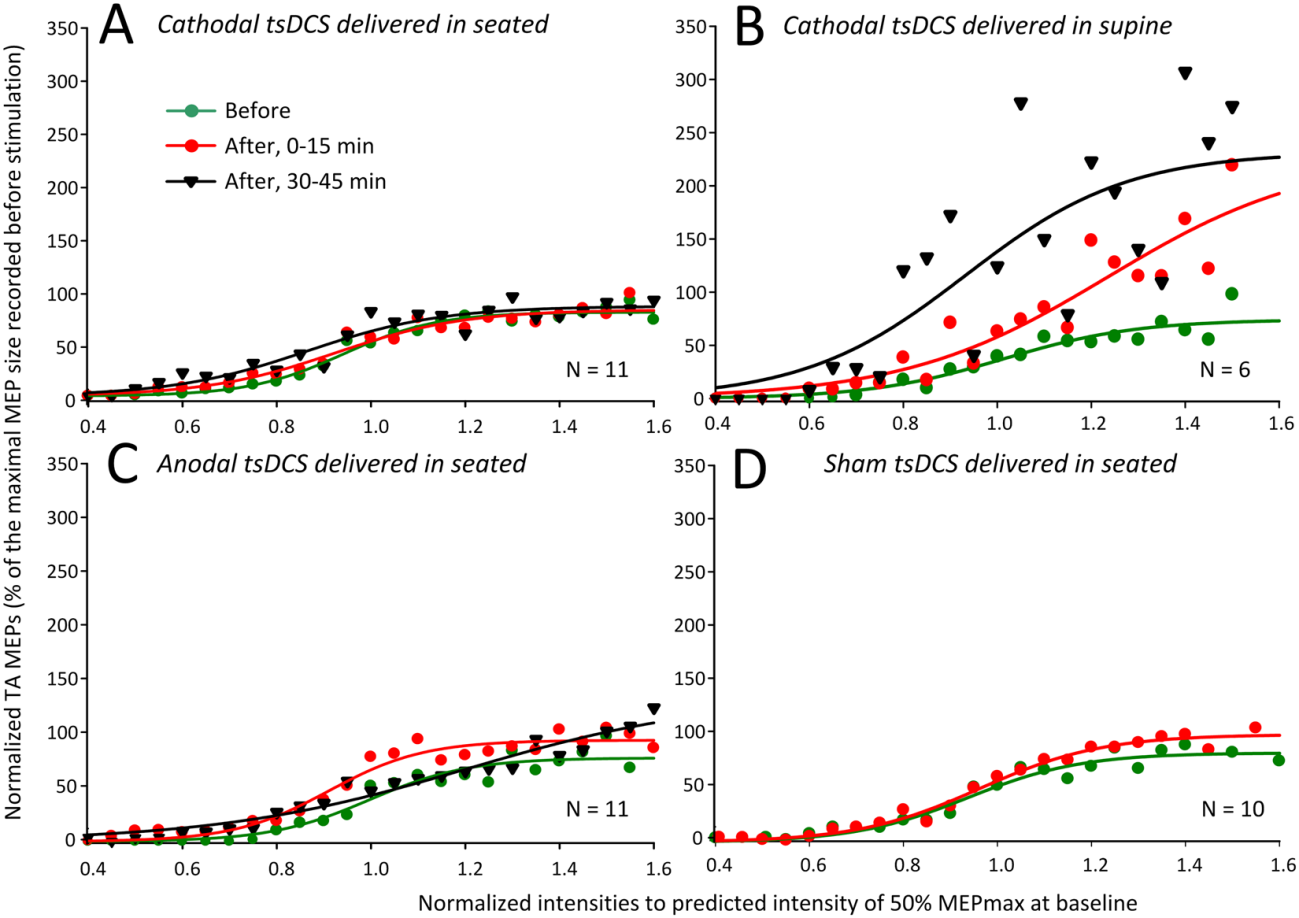
Corticospinal input/output curves before and after transpinal direct current stimulation (tsDCS). Tibialis anterior motor evoked potentials (TA MEPs) input/output curves before (green circles), 0–15 min after (red circles), and 30–45 min after (black triangles) 30 minutes of tsDCS. Cathodal (**A**), anodal (**C**), and sham (**D**) stimulation was delivered whilst the subjects were seated, and cathodal tsDCS (**B**) was delivered with the subjects lying supine. TA MEPs were normalized to the maximal MEP size observed at baseline and plotted in multiples of stimulation intensities corresponding to 50% of the maximal MEP observed at baseline. The sigmoid function fitted to the data is also shown.

Changes in corticospinal input/output function were assessed based on the TA MEP recruitment curves assembled before and after tsDCS at similar stimulation intensities for each subject. The TA MEP input/output curves from all subjects and for all tsDCS polarities tested are indicated in Fig. 3. The TA MEPs are depicted as percentages of the maximal MEP amplitude, and are plotted against the intensities normalized to the predicted stimulation intensity from the sigmoid fit corresponding to the stimulus required to elicit an MEP equivalent to 50% of the maximal MEP (S50-MEPmax) observed before tsDCS.

Two-way rmANOVA with factors time (before, 0–15 min, 30–45 min) and protocol (cathodal seated, cathodal supine, anodal seated, and sham seated) with TA MEPs grouped based on normalized stimulation intensities from 0.4 to 1.2 multiples of S50–MEPmax, showed a significant main effect of time (F_(2)_ = 4.62, *p* = 0.011) and protocol (F_(3)_ = 8.01, *p* < 0.001). Holm-Sidak pairwise multiple comparisons showed that the TA MEP input/output was significantly different between cathodal supine tsDCS and cathodal seated tsDCS (*p* = 0.01), and between cathodal supine tsDCS and anodal seated tsDCS (*p* = 0.009). Further, Holm-Sidak pairwise multiple comparisons across time and all protocols showed that the TA MEP input/output was significantly increased at 0–15 min (*p* = 0.017) and at 30–45 min (*p* < 0.001) compared to that observed before tsDCS. Comparison among protocols showed that the TA MEP input/output was significantly increased after cathodal supine compared to anodal seated (*p* < 0.001), cathodal seated (*p* < 0.001), and sham (*p* < 0.001). These findings suggest that cathodal tsDCS delivered with subjects in the supine position upregulates corticospinal input/output motor function.

### Changes in Spinal Reflex Input/Output Function by tsDCS

**Figure 4:**
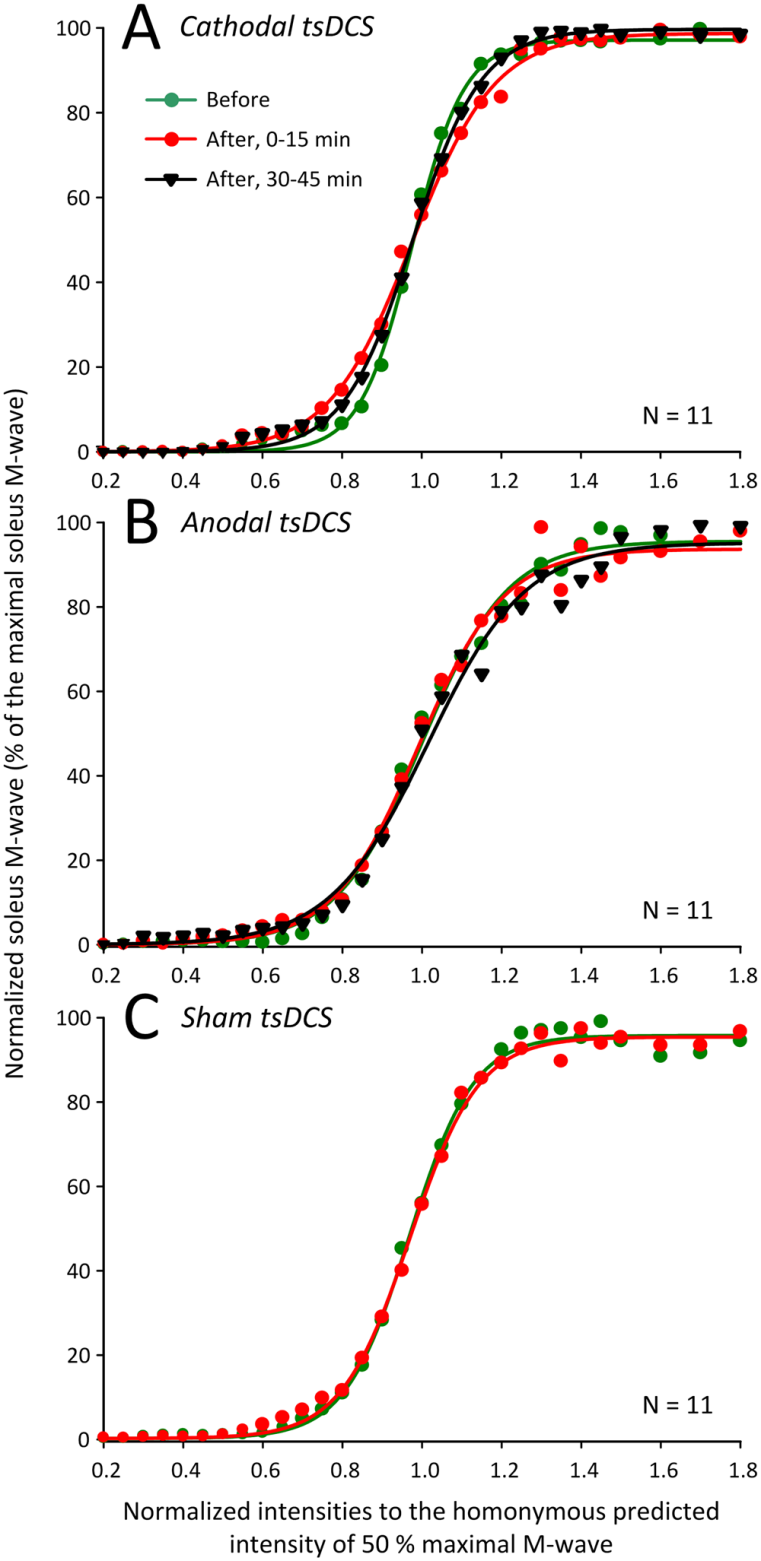
Soleus M-wave input/output curves before and after transpinal direct current stimulation (tsDCS). Soleus M-wave input/output curves from all subjects before (green circles), 0–15 min after (red circles), and 30–45 min after (black triangles) of cathodal (**A**), anodal (**B**), and sham (**C**) tsDCS whilst seated. The soleus M-waves were normalized to the homonymous maximal M-wave and plotted in multiples of stimulation intensities corresponding to the homonymous 50% of the maximal M-wave. The sigmoid function fitted to the data is also shown.

Changes in spinal reflex input/output function based on the soleus H-reflex recruitment curves were assembled at different times before and after tsDCS. The soleus M-wave input/output curves from all subjects assembled before, at 0–15 min, and 30–45 min after cathodal, anodal, and sham tsDCS are shown in Fig. 4. The M-waves before and after tsDCS for all protocols tested were not significantly different, thus any changes on the soleus H-reflex size after tsDCS could not be due to different recruitment patterns of soleus motoneurons by the Ia afferent volleys or changes in excitation of motor axons.

**Figure 5:**
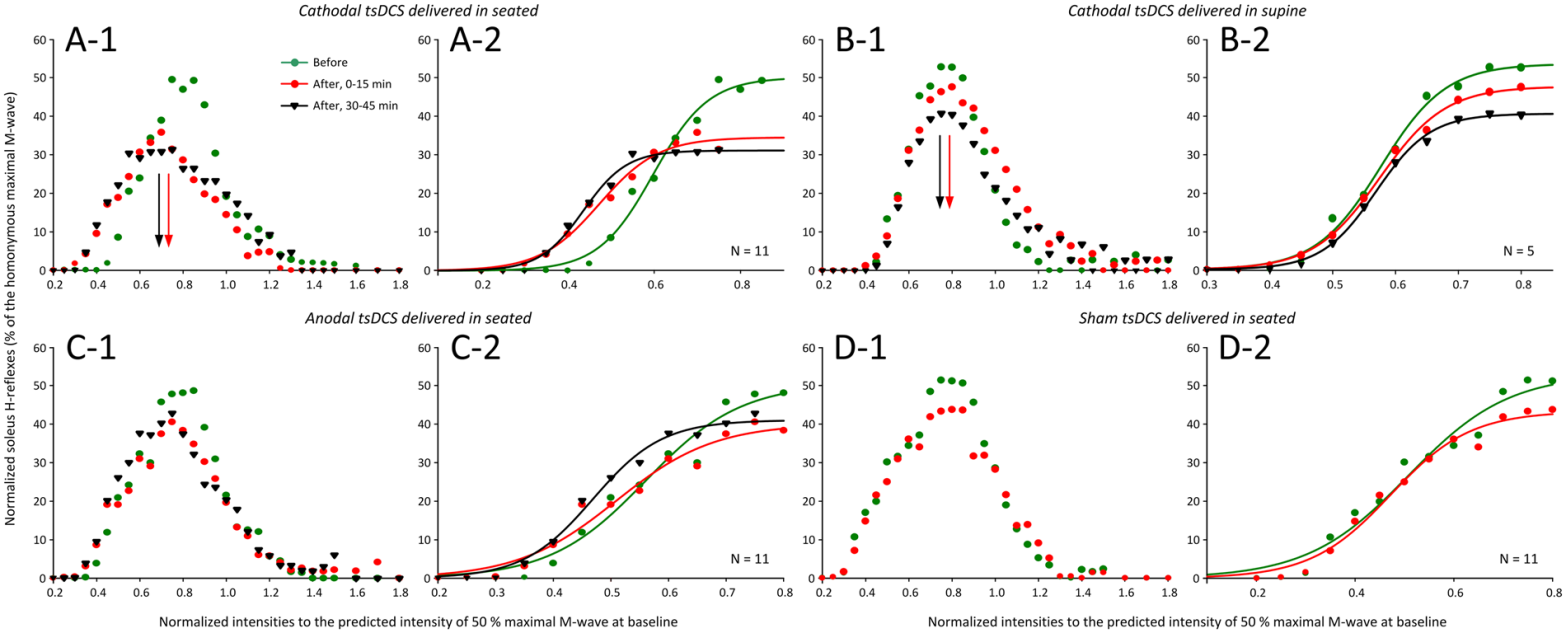
Soleus H-reflex input/output curves before and after transpinal direct current stimulation (tsDCS). Soleus H-reflex input/output curves from all subjects before (green circles), 0–15 min after (red circles), and 30–45 min after (black triangles) 30 minutes of tsDCS. Cathodal (**A-1**), anodal (**C-1**), and sham (**D-1**) stimulation was delivered whilst the subjects were seated and cathodal tsDCS (**B-1**) was delivered with the subjects lying supine. For all protocols the corresponding sigmoid function fitted to the soleus H-reflexes recorded at stimulation intensities that soleus H-reflexes were absent until they reached maximal amplitudes are also shown (**A-2**, **B-2**, **C-2**, **D-2**). Soleus H-reflexes were normalized to the homonymous maximal M-wave and plotted in multiples of stimulation intensities corresponding to 50% of the maximal M-wave observed at baseline.

The soleus H-reflex input/output curves from all subjects assembled before, at 0–15 min, and 30–45 min after cathodal, anodal, and sham tsDCS along with the corresponding sigmoid function fitted at the ascending part of the input/output curve are shown in Fig. 5. Two-way rmANOVA with factors time (before, 0–15 min, 30–45 min) and protocols (cathodal seated, cathodal supine, anodal seated, and sham seated) while the soleus H-reflexes from all subjects were grouped based on the normalized stimulation intensities including 0.05 increments from 0.2 to 0.85 of the stimulus required to elicit an H-reflex equivalent to 50% of the maximal M-wave (S50-Mmax) before tsDCS, showed a significant main effect of time (F_(2)_ = 3.38, *p* = 0.04), and a significant main effect of protocol (F_(3)_ = 30.66, *p* < 0.001). Holm-Sidak pairwise multiple comparisons showed that the soleus H-reflex was significantly different among protocols, with soleus H-reflexes recorded after sham stimulation to be significantly different from all other protocols (*p* < 0.01). In addition, Holm-Sidak pairwise multiple comparisons showed that the soleus H-reflex decreased following cathodal seated as compared to anodal seated tsDCS (*p* = 0.012), and cathodal supine as compared to anodal seated tsDCS (*p* = 0.016), while a significant difference of H-reflexes between cathodal seated and cathodal supine was not found (*p* = 0.12). Further, a leftward shift of the soleus H-reflex input/output curve for the cathodal seated tsDCS was noted (Figs 4A–2). However, the predicted parameters (slope, *m* function of the slope, and stimuli at H-reflex threshold, S50 and S100 of Hmax) estimated for each H-reflex input/output curve separately were not significantly different across time (*p* > 0.05). Thus, we cannot suggest for changes in reflex excitability based on the predicted sigmoid function parameters. Together, these findings suggest that tsDCS decreased soleus H-reflex excitability that depended largely on polarity and not on the body position during which tsDCS was delivered.

## Discussion

In this study, we examined the ability of tsDCS to simultaneously affect cortical feedback neural mechanisms, as well as corticospinal and spinal pathways in healthy individuals. The major findings of this study are that tsDCS affects cortical facilitatory feedback mechanisms, corticospinal input/output function, and spinal reflex function.

Changes in cortical feedback mechanisms were assessed via the well-established paired TMS paradigm delivered at short and medium ISIs^23,24^. At short ISIs, MEP depression has been ascribed to interneuronal circuits in the primary motor cortex^25^, involving largely low-threshold γ-aminobutyric acid receptor-dependent inhibitory pathways^26^. At medium ISIs, MEP facilitation is believed to be nonsynaptic in nature, occurring at the initial axon segment of cortical interneurons involving high-excitatory glutamatergic pathways^24,26–29^.

In this study, we found that the cortical inhibitory profile, regardless of the body position and polarity, did not change after tsDCS (Fig. 2). This finding is in contrast to the decreased cortical inhibition, assessed at 3 ms ISI, estimated from the conditioned MEPs of the first dorsal interosseus and TA muscles after 20 minutes of cathodal supine tsDCS at 2.5 mA^30^. Furthermore, cathodal and anodal tsDCS increased intracortical facilitation when the subjects were seated (Fig. 2A,C), and cathodal tsDCS decreased intracortical facilitation when the subjects were lying supine (Fig. 2B), supporting for a position-dependent effect. As the conditioning TMS pulse was subthreshold minimizing direct activation of cortical motoneurons, we can suggest that modulation of intracortical facilitation may have been mediated by changes of spontaneous activity of motor cortex interneurons based on the documented effects of tsDCS on brain local field potentials and spontaneous activity^31^. However, our findings are in opposition to the unchanged intracortical facilitation, assessed at 10 ms ISI, in arm and leg MEP amplitudes after anodal or cathodal tsDCS^30^. The different results may be related to (1) the duration of which the tsDCS was delivered, 2) strength of baseline cortical facilitation^30^, and 3) strength of tsDCS intensity^5^.

Corticospinal input/output motor function was significantly increased after cathodal tsDCS delivered with subjects lying supine compared to that observed after cathodal or anodal tsDCS delivered in seated (Fig. 3). Corticospinal excitability remained increased at 30–45 min post stimulation only when cathodal tsDCS was delivered with subjects lying supine (Fig. 3B). Based on this finding, we suggest that negative currents are more powerful in eliciting prolonged increases in corticospinal input/output motor function in humans, and that there is a body-position dependency that warrants further investigation. The increased corticospinal excitability observed is in line with the increased MEP amplitudes reported after cathodal and anodal tsDCS delivered over the cervical region^32^, transspinal constant (or pulsed) current stimulation^21^, and tDCS in humans^33^. Studies in animal and human have suggested that tDCS alters the corticospinal drive of spinal motoneurons by influencing the spontaneous firing rate of the corticospinal cells through tonic de- or hyper-polarization of resting membrane potential^34,35^. Based on recent evidence, tsDCS could potentially have altered corticospinal input/output motor output by changing the excitability of myelinated axons within the dorsal columns^36^, polarization of pre-terminal branches of afferent fibers^37^, composition of the TMS-induced indirect (I)-waves^38^, and interhemispheric inhibition^39^.

In contrast to the facilitatory effects we observed on corticospinal excitability, soleus H-reflex excitability was decreased after cathodal tsDCS regardless of the body position and remained unaffected after anodal or sham tsDCS (Fig. 5). When considered in light of other studies, these findings suggest that tsDCS affects spinal input/output reflex function of arms and legs differently, since cervical cathodal tsDCS increased H-reflex excitability and cervical anodal tsDCS had the exact opposite effect^40^. Non-significant effects on H-reflex excitability or spinal inhibitory mechanisms of the arms after cervical tsDCS have also been reported^41^, supporting for large variability among research studies. Furthermore, moderate effects of tsDCS on soleus H-reflex input/output curves^15^ along with unchanged Hmax/Mmax ratios^14,30^ have been reported. While there is large variability among studies with respect to electrode dimensions and position, subject posture during current delivery, and the duration and intensity of stimulation, assumptions based solely on the maximal H-reflex amplitude need to be viewed cautiously as the maximal H-reflex is non-monosynaptic, and its size is determined by the excitability state of complex spinal interneuronal circuits^42,43^.

Possible mechanisms for the effects of cathodal tsDCS on soleus H-reflex excitability are membrane potential changes of primary group Ia afferent fibers^44^. It is anticipated that tsDCS over the thoracic region may activate cutaneous afferents, which are known to affect Ia afferent discharges and alter both corticospinal and spinal reflex excitability^45,46^. However, it is unlikely that cutaneous afferent activation contributed to the observed effects because cutaneous perception is similar regardless of the current polarity^47^. Modeling studies concerning transspinal stimulation have suggested that current density is maximal at the intervertebral spaces through which spinal ventral and dorsal roots exit the spinal cord^48^. Because current density and thus neural elements being excited by tsDCS depends on the position of the reference electrode^49^, human modeling studies are needed to delineate the neural elements being stimulated and the exact neurophysiological mechanisms by which tsDCS acts on spinal neural circuits.

In conclusion, the present study provides convincing evidence that 30 minutes of tsDCS can change remote motor pathways. tsDCS findings are promising and may be beneficial for enhancing motor cortex output in neurological disorders. Thus far, the plastic changes observed with tDCS or tsDCS have been transient and reversible. Our findings suggest that neural excitability changes last or even increase after 30 minutes of stimulation offset. However, repetitive application of tsDCS on cortical, corticospinal, and spinal reflex excitability or postulation of permanent changes with this method needs further investigation. Lastly, pairing tsDCS with other neuromodulatory methods might be a solution to potentiate the neurophysiological changes in neurological disorders.

### Limitations of the Study

In this study, we recorded MEPs and H-reflexes in response to four different polarities/body positions; cathodal seated, cathodal supine, anodal seated and sham. However, sham was only delivered in the seated position and not in the supine position. Secondly, consecutive test sessions were performed at a 4-week interval. Because the effects after a single DC stimulation session last up to a few hours after current offset^50,51^, the interval among sessions was sufficient for all neural changes due to tsDCS to have been abolished. Third, because the number of MEPs being acquired affects their reliability^52^, and baseline MEP variability is related to neuroplasticity protocols^53^, large scale studies incorporating I-wave protocols, which are known to contribute to baseline MEP variability, are needed. Optimal stimulation intensities for changes of corticospinal and spinal motor pathways following tsDCS also warrant further investigation, since anodal and cathodal tDCS effects are intensity-dependent manifested in a non-linear fashion^54^.

## Methods

### Subjects

For all twenty-two healthy subjects, eligibility to the study was established based on a TMS safety screening questionnaire. Written informed consent was obtained prior to study enrollment, and all experimental procedures were conducted in compliance with the Declaration of Helsinki after Institutional Review Board approval by the City University of New York. None of the subjects were taking medication that affect neural excitability or had a history of neurological or musculoskeletal disorder. All subjects but one was right leg dominant, and all were naïve to tsDCS. A total of 42 experiments were completed on different days. Sixteen subjects participated in cathodal, anodal and/or sham tsDCS delivered whilst seated; however due to lack of availability only nine of these subjects were able to complete all three protocols. An additional six subjects participated in the cathodal tsDCS protocol delivered whilst lying supine. All recordings were conducted with a 4-week interval between protocols. Due to a large varicose vein, one of these subjects was tested on their left leg for the soleus H-reflex and M-wave input/output curve. Blood pressure of all participants was monitored periodically during the experiment and no significant changes were noted.

### EMG recordings

Surface electromyography (EMG) was recorded by single bipolar differential electrodes (MA300–28, Motion Lab Systems Inc., Baton Rouge, LA) from the right TA and soleus muscles. EMG signals were amplified, filtered (10–1000 Hz), sampled at 5000 Hz via a 1401 plus data acquisition interface (Cambridge Electronics Design Ltd., England, UK), and stored for offline analysis.

### tsDCS for neuromodulation

tsDCS was applied through a rubber pad saline-soaked sponge electrode (3.2 cm × 3.2 cm; Amrex Electrode, USA), and due to its size it covered from Thoracic 10 to Thoracic 12 vertebral levels. These vertebral levels correspond to Lumbar 1–4 spinal segments and thus to the segmental innervation of the muscles from which compound muscle action potentials were recorded in this study. It should also be noted that with the active stimulating electrode we were targeting the spinal cord ensuring also that cauda equina was not stimulated. Stimulation was delivered via a battery-driven direct current stimulator (neuroConn DC stimulator plus, Germany) with subjects in the seated or supine position. The spinal electrode determined the polarity of tsDCS. For cathodal and anodal polarities, stimulation was delivered for 30 minutes and intensity was set at 4.0 mA. The reference electrode (a rubber pad saline-soaked sponge electrode 10.16 cm × 10.16 cm; Amrex Electrode, USA) was positioned over the thigh along the sciatica nerve. The resulted current density was 0.39 mA/cm^2^, which is consistent with current densities (above 25 mA/cm^2^) that do not induce brain tissue damage even at high-frequency stimulation over several hours^55,56^. Further, the total charge was 7.03125 C/cm^2^, well below the harmful range, supporting the need for more intensified tsDCS protocols^56^. Sham stimulation was delivered via a pre-programmed protocol to mimic real stimulation by delivering 1 mA for one minute followed by small current pulses every 550 ms as an impedance control.

### Neurophysiological Tests

The neurophysiological tests described below were conducted before, immediately after (0–15 min), and 30–45 min after tsDCS in a randomized order. Stimulation intensities remained unchanged across time for each subject during TMS stimulation protocols.

### Cortical excitability

Changes in cortical excitability were assessed by establishing changes in short-latency intracortical inhibition and medium-latency intracortical facilitation. Right TA MEPs in response to paired TMS pulses over the left primary cortex (Magstim BiStim^2^, UK) at different short ISIs of 1, 2, 3 and medium ISIs of 15, 20, and 25 ms were recorded randomly with the unconditioned (or control) MEPs. Conditioned MEPs at these intervals represent a measure of plasticity in the motor cortex because intracortical inhibition and intracortical facilitation do not depend on changes in spinal excitability^24,57^. In the seated cathodal tsDCS protocol, the conditioning (first stimulus) and test (second stimulus) TMS pulses were set at 0.76 ± 0.06 (34.6 ± 6.26 maximum stimulator output (MSO); mean ± SD) and 1.24 ± 0.04 (56.3 ± 7.5 MSO) TA MEP resting threshold across subjects, respectively. In the seated anodal tsDCS protocol, the conditioning and test TMS pulses were set at 0.77 ± 0.07 (34 ± 6.6 MSO) and 1.30 ± 0.08 (57 ± 10.7 MSO) TA MEP resting threshold across subjects, respectively. Adjustments of TMS intensities were made in response to discomfort reported by few subjects resulting in unequal multiples of TMS intensities as multiples of MEP threshold between protocols. Similar ranges of TMS intensities were used for supine cathodal and seated sham tsDCS. Under control conditions, 24 MEPs at test pulse intensities were recorded at 0.1 Hz. Following subthreshold conditioning TMS, 12 MEPs at 0.1 Hz were recorded at each ISI.

### Corticospinal excitability

Changes in corticospinal excitability were assessed based on the right TA MEP input/output curves. Input/output curves were assembled in ascending order from stimulation intensities corresponding to 0.4 TA MEP resting threshold until maximum amplitudes were obtained. At least 4 MEPs at 0.1 Hz were recorded at different stimulation intensities.

### Spinal excitability

Changes in spinal excitability were assessed based on the right soleus H-reflex and M-wave input/output curves, except for one performed on the left. With the subject seated and maintaining a 120° knee angle, a stainless steel plate of 4 cm^2^ in diameter (anode) was secured proximal to the patella of the right leg and connected to a constant current stimulator (DS7A, Digitimer, UK). A rectangular single pulse stimulus of 1-ms duration triggered by Spike 2 scripts (CED Ltd., UK) was delivered to the posterior tibial nerve in the popliteal fossa via a hand-held monopolar stainless steel head electrode (cathode) used as a probe. The optimal stimulation site was defined as the site that elicited an M-wave of similar shape to that of the H-reflex at low and high stimulation intensities, and at the lowest stimulation intensity an H-reflex could be evoked without an M-wave. When the optimal site was identified, the monopolar electrode was replaced by a pre-gelled disposable electrode (SureTrace, Conmed, Utica, NY, USA) and position was visually reconfirmed. Electrodes were maintained under constant pressure throughout the experiment. Soleus H-reflexes and M-waves were then recorded from low (absent responses) to high stimulation intensities where only the M-wave was present and remained unchanged despite further increments in stimulus intensity.

### Data analysis and statistics

All waveforms were measured as the area of the fullwave rectified EMG signal (Spike 2, CED Ltd., UK). Unconditioned TA MEPs were used to measure latency based on the cumulative sum technique on the rectified waveform average^58^, where latency corresponds to the first positive deflection point above background EMG activity measured from 60 ms of pre-stimulus EMG activity. All data were subjected to the Shapiro-Wilk test for normal distribution.

For each subject and stimulation protocol (cathodal seated, cathodal supine, anodal seated, vs. sham seated) over time (before, 0–15 min, 30–45 min), the TA MEPs evoked upon paired TMS pulses at different ISIs (short ISIs of 1, 2, 3 and medium ISIs of 15, 20, and 25 ms) were measured and normalized to the mean amplitude of the homonymous unconditioned MEP. The normalized average conditioned MEP amplitude from each subject was grouped based on time of testing, stimulation protocols (including sham), and ISIs. A two-way ANOVA was performed to establish the main effects of time and protocol with the conditioned TA MEPs grouped based on the ISI, separately for short and medium ISIs. When a statistically significant main effect was found, Holm-Sidak *t*-tests for multiple comparisons were used to test for significant interaction effects across time and between protocols.

For data comprising the soleus H-reflex or TA MEP input/output curves from each subject, a Boltzmann sigmoid function (Eq. 1; SigmaPlot 11, Systat Software Inc.) was fitted to the averages plotted against the stimulation intensities. The estimated parameters in ‘equation (1)’ denote the Hmax or MEPmax, the slope parameter of the function (m), the S50-Mmax or S50-MEPmax, and the H-reflex or MEP amplitude at a given stimulus value H(s). The slope and stimuli corresponding to threshold and maximal amplitudes were calculated based on equations (2), (3) and (4), respectively.

1 $${\rm{MEP}}({\rm{s}})=\frac{{\rm{MEPmax}}}{(1+\exp ({\rm{m}}({\rm{s}}50-{\rm{s}})))\,}\,\,{\rm{H}}({\rm{s}})=\,\frac{{\rm{Hmax}}}{(1+\exp ({\rm{m}}({\rm{S}}50-{\rm{s}})))\,}$$

2 $${\rm{MEPslope}}=\frac{{\rm{m}}\times {\rm{MEPmax}}}{4}\,\,{\rm{Hslope}}={\rm{m}}\frac{{\rm{Hmax}}}{4}$$

3 $$\mathrm{MEPth}\,\mathrm{stim}=\frac{{\rm{s}}-2}{{\rm{m}}}\,\,{\rm{Hth}}=\frac{{\rm{s}}-2}{{\rm{m}}}$$

4 $$\mathrm{MEPmax}\,\mathrm{stim}=\frac{{\rm{s}}+2}{{\rm{m}}}\,\,{\rm{Hmax}}=\frac{{\rm{s}}+2}{{\rm{m}}}$$

The S50–MEPmax at baseline was used to normalize the TMS intensities across time in order for the MEP input/output curves to be grouped across subjects, while the TA MEPs were normalized to the MEPmax amplitude recorded at baseline. The average normalized MEP size was calculated in increments of 0.05 multiples of S50–MEPmax for each subject and across subjects. The average normalized MEP across subjects was subjected to a two-way rmANOVA to establish the main effects of time and stimulation protocols, including also sham. When a statistically significant effect was found, Holm-Sidak *t*-tests for multiple comparisons were used to test for significant interactions across time and between protocols.

For each subject, the soleus M-waves, measured as the area under the fullwave rectified curve, recorded at varying stimulation intensities were normalized to the homonymous Mmax amplitude to counteract for differences of muscle geometry across subjects^59^, and were plotted against the actual stimulation intensities. A Boltzmann sigmoid function (Eq. 1) was then fitted to the full soleus M-wave input/output curve^60–62^. This was done separately for each individual input/output curve. The predicted S50-Mmax was derived from the sigmoid fit and subsequently used to normalize the stimulation intensities that evoked each soleus M-wave^62^. Averages of normalized M-waves were calculated in steps of 0.05 (up to 1.0 times the S50-Mmax) and 0.1 ( > 1.0 times the S50–Mmax) for each subject and grouped based on time, polarity, and multiples of stimulation intensities across subjects.

A similar analysis was also performed for the soleus H-reflex, but the stimulation intensities that the soleus H-reflexes were recorded before, at 0–15 min and at 30–45 min after tsDCS were normalized to S50–Mmax at baseline. The average normalized soleus H-reflex was calculated in increments of 0.05 multiples of S50–Mmax for each subject and across subjects. The average normalized soleus H-reflex across subjects was subjected to a two-way rmANOVA to establish the main effects of time and protocol. When a statistically significant effect was found, Holm-Sidak *t*-tests for multiple comparisons were used to test for significant main effects of time and stimulation protocol.

### Data availability

The datasets generated during and/or analyzed during the current study are available from the corresponding author on reasonable request.

## References

[1] Priori A, Ciocca M, Parazzini M, Vergari M, Ferrucci R (2014). Transcranial cerebellar direct current stimulation and transcutaneous spinal cord direct current stimulation as innovative tools for neuroscientists. J. Physiol..

[2] Nitsche MA, Paulus W (2000). Excitability changes induced in the human motor cortex by weak transcranial direct current stimulation. J. Physiol..

[3] Jeffery DT, Norton JA, Roy FD, Gorassini MA (2007). Effects of transcranial direct current stimulation on the excitability of the leg motor cortex. Exp. Brain. Res..

[4] Galea JM, Jayaram G, Ajagbe L, Celnik P (2009). Modulation of cerebellar excitability by polarity-specific noninvasive direct current stimulation. J. Neurosci..

[5] Batsikadze G, Moliadze V, Paulus W, Kuo MF, Nitsche MA (2013). Partially non-linear stimulation intensity-dependent effects of direct current stimulation on motor cortex excitability in humans. J. Physiol..

[6] Carter MJ, Maslovat D, Carlsen AN (2015). Anodal transcranial direct current stimulation applied over the supplementary motor area delays spontaneous antiphase-to-in-phase transitions. J. Neurophysiol..

[7] Roche N, Lackmy A, Achache V, Bussel B, Katz R (2009). Impact of transcranial direct current stimulation on spinal network excitability in humans. J. Physiol..

[8] Roche N, Lackmy A, Achache V, Bussel B, Katz R (2011). Effects of anodal transcranial direct current stimulation over the leg motor area on lumbar spinal network excitability in healthy subjects. J. Physiol..

[9] Liebetanz D, Nitsche MA, Tergau F, Paulus W (2002). Pharmacological approach to the mechanisms of transcranial DC-stimulation-induced after-effects of human motor cortex excitability. Brain.

[10] Cogiamanian F, Vergari M, Pulecchi F, Marceglia S, Priori A (2008). Effect of spinal transcutaneous direct current stimulation on somatosensory evoked potentials in humans. Clin. Neurophysiol..

[11] Cogiamanian F (2011). Transcutaneous spinal cord direct current stimulation inhibits the lower limb nociceptive flexion reflex in human beings. Pain.

[12] Nierat MC, Similowski T, Lamy JC (2014). Does trans-spinal direct current stimulation alter phrenic motoneurons and respiratory neuromechanical outputs in humans? A double-blind, sham-controlled, randomized, crossover study. J. Neurosci..

[13] Bocci T (2015). Transcutaneous spinal direct current stimulation modulates human corticospinal system excitability. J. Neurophysiol..

[14] Winkler T, Hering P, Straube A (2010). Spinal DC stimulation in humans modulates post-activation depression of the H-reflex depending on current polarity. Clin. Neurophysiol..

[15] Lamy JC, Ho C, Badel A, Arrigo RT, Boakye M (2012). Modulation of soleus H reflex by spinal DC stimulation in humans. J. Neurophysiol..

[16] Bocci T (2014). Cathodal transcutaneous spinal direct current stimulation (tsDCS) improves motor unit recruitment in healthy subjects. Neurosci. Lett..

[17] Kaczmarek D, Ristikankare J, Jankowska E (2017). Does trans-spinal and local DC polarization affect presynaptic inhibition and post-activation depression?. J. Physiol..

[18] Bolzoni F, Jankowska E (2015). Presynaptic and postsynaptic effects of local DC polarization within the spinal cord in anaesthetized animal preparations. J. Physiol..

[19] Knikou M (2013). Neurophysiological characterization of transpinal evoked potentials in human leg muscles. Bioelectromagnetics.

[20] Knikou M (2014). Transpinal and transcortical stimulation alter corticospinal excitability and increase spinal output. PLoS One.

[21] Knikou M, Dixon L, Santora D, Ibrahim MM (2015). Transspinal constant-current long-lasting stimulation: a new method to induce cortical and corticospinal plasticity. J. Neurophysiol..

[22] Danner SM (2016). Body position influences which neural structures are recruited by lumbar transcutaneous spinal cord stimulation. PLoS One.

[23] Kujirai T (1993). Corticocortical inhibition in human motor cortex. J. Physiol..

[24] Di Lazzaro V (1998). Magnetic transcranial stimulation at intensities below active motor threshold activates intracortical inhibitory circuits. Exp. Brain. Res..

[25] Ziemann U, Rothwell JC, Ridding MC (1996). Interaction between intracortical inhibition and facilitation in human motor cortex. J. Physiol..

[26] Ilic TV (2002). Short-interval paired-pulse inhibition and facilitation of human motor cortex: the dimension of stimulus intensity. J. Physiol..

[27] Di Lazzaro V (2007). Segregating two inhibitory circuits in human motor cortex at the level of GABAA receptor subtypes: a TMS study. Clin. Neurophysiol..

[28] Liepert J, Schwenkreis P, Tegenthoff M, Malin JP (1997). The glutamate antagonist riluzole suppresses intracortical facilitation. J. Neural Transm..

[29] Ziemann U (2003). Pharmacology of TMS. Suppl. Clin. Neurophysiol..

[30] Bocci T (2015). Spinal direct current stimulation modulates short intracortical inhibition. Neuromodulation.

[31] Aguilar J (2011). Spinal direct current stimulation modulates the activity of gracile nucleus and primary somatosensory cortex in anaesthetized rats. J. Physiol..

[32] Lim CY, Shin HI (2011). Noninvasive DC stimulation on neck changes MEP. Neuroreport.

[33] Wiethoff S, Hamada M, Rothwell JC (2014). Variability in response to transcranial direct current stimulation of the motor cortex. Brain Stimul..

[34] Bindman LJ, Lippold OC, Redfearn JW (1964). The action of brief polarizing currents on the cerebral cortex of the rat (1) during current flow and (2) in the production of long-lasting after-effects. J. Physiol..

[35] Creutzfeldt OD, Fromm GH, Kapp H (1962). Influence of transcortical d-c currents on cortical neuronal activity. Exp. Neurol..

[36] Jankowska E, Kaczmarek D, Bolzoni F, Hammar I (2017). Long-lasting increase in axonal excitability following epidurally applied DC. J. Neurophysiol. jn..

[37] Jankowska E, Kaczmarek D, Bolzoni F, Hammar I (2016). Evidence that some long-lasting effects of direct current in the rat spinal cord are activity-independent. Eur. J. Neurosci..

[38] Lang N (2011). Transcranial direct current stimulation effects on I-wave activity in humans. J. Neurophysiol..

[39] Bocci T (2015). An unexpected target of spinal direct current stimulation: Interhemispheric connectivity in humans. J Neurosci Methods.

[40] Song W, Amer A, Ryan D, Martin JH (2016). Combined motor cortex and spinal cord neuromodulation promotes corticospinal system functional and structural plasticity and motor function after injury. Exp. Neurol..

[41] Dongés SC, D’Amico JM, Butler JE, Taylor JL (2017). The effects of cervical transcutaneous spinal direct current stimulation on motor pathways supplying the upper limb in humans. PLoS One.

[42] Burke D, Gandevia SC, McKeon B (1984). Monosynaptic and oligosynaptic contributions to human ankle jerk and H-reflex. J. Neurophysiol..

[43] Knikou M (2008). The H-reflex as a probe: pathways and pitfalls. J. Neurosci. Methods.

[44] Eccles JC, Kostyuk PG, Schmidt RF (1962). The effect of electric polarization of the spinal cord on central afferent fibres and on their excitatory synaptic action. J. Physiol..

[45] Delwaide PJ, Crenna P (1983). Exteroceptive influences on lower limb motoneurons in man: spinal and supraspinal contributions. Adv. Neurol..

[46] Mackey AS, Uttaro D, McDonough MP, Krivis LI, Knikou M (2016). Convergence of flexor reflex and corticospinal inputs on tibialis anterior network in humans. Clin. Neurophysiol..

[47] Ambrus GG, Antal A, Paulus W (2011). Comparing cutaneous perception induced by electrical stimulation using rectangular and round shaped electrodes. Clin. Neurophysiol..

[48] Parazzini M (2014). Modeling the current density generated by transcutaneous spinal direct current stimulation (tsDCS). Clin. Neurophysiol..

[49] Priori A, Ciocca M, Parazzini M, Vergari M, Ferrucci R (2014). Transcranial cerebellar direct current stimulation and transcutaneous spinal cord direct current stimulation as innovative tools for neuroscientists. J. Physiol..

[50] Nitsche MA, Paulus W (2001). Sustained excitability elevations induced by transcranial DC motor cortex stimulation in humans. Neurology.

[51] Nitsche MA (2008). Transcranial direct current stimulation: state of the art. Brain Stim..

[52] Hashemirad F, Zoghi M, Fitzgerald PB, Jaberzadeh S (2017). Reliability of motor evoked potentials induced by transcranial magnetic stimulation: The effects of initial motor evoked potentials removal. Basic Clin. Neurosci..

[53] Hordacre B (2017). Variability in neural excitability and plasticity induction in the human cortex: A brain stimulation study. Brain Stimul..

[54] Jamil A (2017). Systematic evaluation of the impact of stimulation intensity on neuroplastic after-effects induced by transcranial direct current stimulation. J. Physiol..

[55] McCreery DB, Agnew WF, Bullara LA, Yuen TG (1990). Partial pressure of oxygen in brain and peripheral nerve during damaging electrical stimulation. J Biomed Eng.

[56] Liebetanz D (2009). Safety limits of cathodal transcranial direct current stimulation in rats. Clin. Neurophysiol..

[57] Chen R (1998). Intracortical inhibition and facilitation in different representations of the human motor cortex. J. Neurophysiol..

[58] Ellaway PH (1978). Cumulative sum technique and its application to the analysis of peristimulus time histograms. Electroencephalogr. Clin. Neurophysiol..

[59] Pierrot-Deseilligny, E. & Burke, D. The Circuitry of the Human Spinal Cord. Cambridge University Press, 2005.

[60] Brinkworth RS, Tuncer M, Tucker KJ, Jaberzadeh S, Türker KS (2007). Standardization of H-reflex analyses. J. Neurosci. Methods.

[61] Klimstra M, Zehr EP (2008). A sigmoid function is the best fit for the ascending limb of the Hoffmann reflex recruitment curve. Exp. Brain. Res..

[62] Smith AC, Rymer WZ, Knikou M (2015). Locomotor training modifies soleus monosynaptic motoneuron responses in human spinal cord injury. Exp. Brain Res..

